# Double-Outlet Right Ventricle in a Chianina Calf

**DOI:** 10.3390/ani11020318

**Published:** 2021-01-27

**Authors:** Domenico Caivano, Maria Chiara Marchesi, Piero Boni, Noemi Venanzi, Giovanni Angeli, Francesco Porciello, Elvio Lepri

**Affiliations:** 1 Department of Veterinary Medicine, University of Perugia, Via San Costanzo 4, 06126 Perugia, Italy; noemi.venanzi@studenti.unipg.it (N.V.); giovanni.angeli@unipg.it (G.A.); francesco.porciello@unipg.it (F.P.); elvio.lepri@unipg.it (E.L.); 2 Veterinary Practice, Via Martiri di Modena 2, 06033 Perugia, Italy; pieroboni@libero.it

**Keywords:** cattle, congenital malformation, echocardiography, heart

## Abstract

**Simple Summary:**

This case report describes a rare complex congenital cardiac malformation in a Chianina calf referred to the Veterinary Teaching Hospital of Perugia University (Italy). We describe the clinical, ultrasonographic and pathological findings of a double-outlet right ventricle associated with interventricular and interatrial septal defects. These congenital cardiac anomalies have never been reported in the Chianina breed.

**Abstract:**

Congenital heart defects have been occasionally reported in cattle and ventricular septal defect represents the most frequently encountered anomaly. The double-outlet right ventricle is a rare congenital ventriculoarterial malformation reported only in certain cattle breeds. We describe this rare and complex congenital cardiac malformation observed in a 10-day-old male Chianina calf. Clinical examination showed tachycardia, tachypnea, jugular pulses, cyanotic mucous membranes and a right apical systolic murmur. Transthoracic echocardiography revealed severe dilation of the right-sided cardiac chambers with a markedly hypoplastic left ventricle. Both aorta and pulmonary artery leaving the right ventricle in parallel alignment with the tricuspid valve were suggestive of a dual-outlet right ventricle. Interventricular and interatrial septal defects were also visualized. Post-mortem examination confirmed the echocardiographic findings. To the authors’ knowledge, a similar complex congenital cardiac malformation has not been reported in calves of the Chianina breed to date.

## 1. Introduction

Congenital heart defects (CHD) are occasionally reported in cattle with an incidence of 0.7%. Most bovine CHD are simple malformations and ventricular septal defect (VSD) represents the most frequently encountered anomaly [1,2,3,4]. Atrial septal defects (ASD) are reported as “rare” or “common” based on different reports: Murakami et al. (1991) described 44 ASD on 243 malformed bovine hearts [5]. Complex cardiac malformations, as the conotruncal anomalies (tetralogy of Fallot, transposition of the great vessels, double-outlet right ventricle) have been reported anecdotally. The double-outlet right ventricle (DORV) is a rare congenital ventriculoarterial malformation where both arterial trunks emerge from the right ventricle. This complex cardiac anomaly has been described only in certain cattle breeds [6,7,8,9,10], but it has never been reported in the Chianina breed.

The Chianina is one of the oldest cattle breeds in the world, originally raised for draught, that was successively selected for its high-quality meat (the famous “Florentine steak”). The Chianina breed is an Italian breed that originates in central Italy, but that is also present outside of Italy, mainly in South America. Despite its widespread geographic distribution, scientific literature is scarce about congenital diseases in this cattle breed, with reports of congenital pseudomyotonia [11,12,13,14] and ichthyosis [15]. Prevalence of congenital pseudomyotonia carriers and frequency of deleterious allele have been reported in selected subpopulations (13.6% and 13.4%, respectively) [13].

Therefore, the aim of this case report is to describe the clinical presentation, imaging findings and postmortem examination findings in a Chianina calf affected by DORV with interatrial and interventricular communications.

## 2. Materials, Methods and Results

### 2.1. Case Description and Clinical Investigations

A 10-day-old, 40 kg, male Chianina calf was presented to the Veterinary Teaching Hospital of Perugia University for evaluation of dyspnea, lethargy and progressive recumbency. All procedures were performed after obtaining written consent from the calf’s owner. At presentation, the calf showed a temperature of 38.5 °C, tachycardia with regular heart rate (170 beats per minute), tachypnea with respiratory rate of 60 breaths per minute, jugular pulses and cyanotic mucous membranes, especially evident when he was coaxed to move for a short distance. His heartbeat was stronger on the right sided precordial area than the left. Thoracic auscultation revealed harsh bronchovesicular sounds and a grade IV/VI right apical systolic murmur. Umbilicus, peripheral lymph nodes, and joints of all four limbs were normal. Electrocardiogram showed a sinus tachycardia with a heart rate of 170 beats for minute.

**Figure 1:**
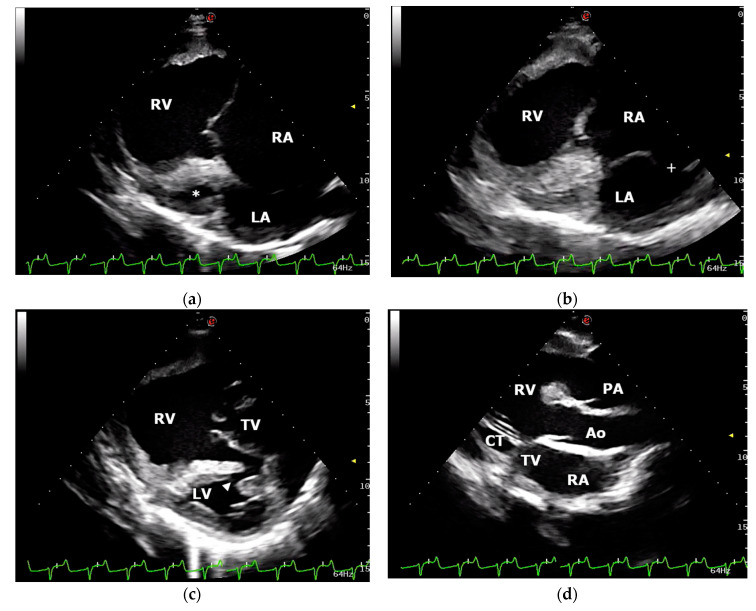
Echocardiographic examination: (**a**) right parasternal, long-axis, four-chamber view demonstrating the severely dilated right atrium (RA) and ventricle (RV) and the markedly hypoplastic left ventricle (*); (**b**) right parasternal, long-axis, four-chamber view showing interatrial septal defect (**+**); (**c**) right parasternal, long-axis, four-chamber view optimized to visualize a small muscular interventricular defect (arrowhead); (**d**) left cranial parasternal, long-axis, oblique view showing aorta (Ao) and pulmonary artery (PA) leaving the RV in parallel alignment with the tricuspid valve (TV). LA, left atrium; LV, left ventricle; CT, chordae tendineae.

Transthoracic echocardiography was performed using an ultrasound unit equipped with multifrequency 1–4 MHz phased-array transducer (MyLab Class C, Esaote, Genova, Italy) and revealed severe dilation of the right ventricle and atrium with a markedly hypoplastic left ventricle (Figure 1). End-diastolic and end-systolic right ventricular (RV) diameters measured using the two-dimensional guided M-mode from the right parasternal ventricular short-axis view were 64.6 mm and 49.3, respectively. The RV fractional area change (FAC) was 51% (FAC = (RVAd–RVAs/RVAd) × 100, where RVAd/RVAs were RV end-diastolic and end-systolic area, respectively). The mitral valve apparatus showed a severely reduced mitral valve orifice. Interatrial septum presented an excessive mobility during the cardiac cycle and a large interatrial septal defect (16 mm) localized in the upper portion of the septum was visualized (Figure 1). Long- and short-axis echocardiographic views of the interventricular septum allowed the visualization of a small muscular interventricular defect (6 mm) localized in the dorsal part of the muscular portion of the septum (Figure 1). An oblique left cranial parasternal long-axis view revealed two great vessels leaving the right ventricle in parallel alignment with the tricuspid valve (Figure 1). The aorta and pulmonary artery were differentiated by identifying the coronary arteries arising from the aorta, and the left and right pulmonary arteries arising from the main pulmonary artery. Color-flow Doppler demonstrated severe tricuspid valve regurgitation and confirmed shunting of the blood through the interventricular and interatrial communications. Agitated saline contrast echocardiography performed through a catheter in the left jugular vein allowed visualization of the bubbles in the left atrium and ventricle. Moreover, bubbles were noted leaving the right ventricle via aorta and pulmonary artery simultaneously. A diagnosis of DORV with interatrial and interventricular septal defects was made. Based on the severity of the clinical signs, echocardiographic findings, and prognosis, euthanasia was chosen by the calf’s owner. After sedation, the calf was euthanized with barbiturate solution intravenously.

### 2.2. Gross Examination and Histopathology

**Figure 2:**
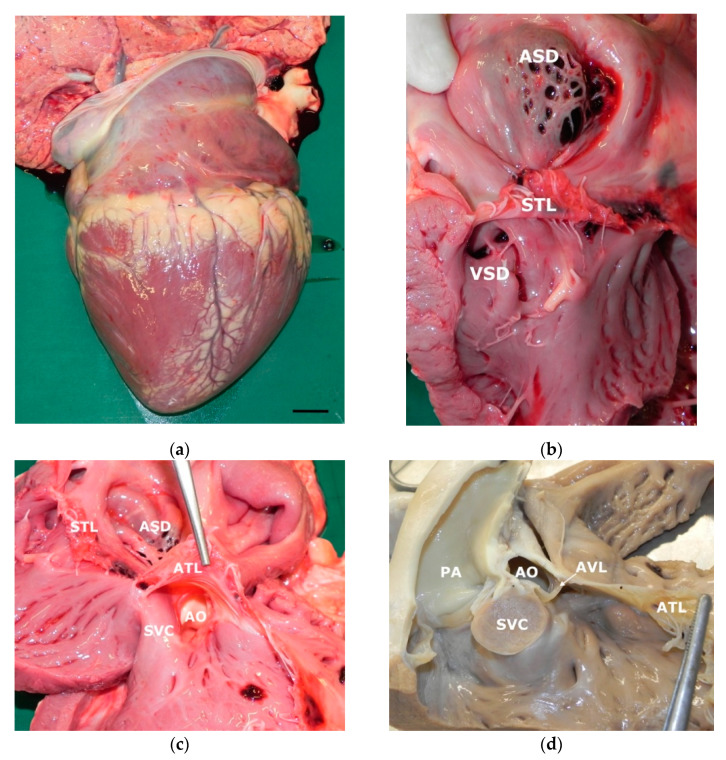
Gross findings in the heart: (**a**) very enlarged right ventricle and atrium (Bar: 2 cm); (**b**) open view of right ventricle and atrium: a large ostium secundum-type atrial septal defect (ASD) is visible in the upper portion of interatrial septum; in the ventricle double paired small ventricular septal defects (VSD) are present in the muscular portion of ventricular septum just beneath the septal tricuspidal leaflet (STL); (**c**) open view of right ventricle inflow tract: aortic ostium (AO) is placed between the supraventricular crest (SVC) and the anterior tricuspidal leaflet (ATL); (**d**) right ventricle outflow tract after cutting the supraventricular crest: the pulmonary artery (PA) and aorta arise from right ventricle very close each other. AVL: aortic valve leaflet.

A complete post mortem examination was performed on the right side recumbency. At the opening of the abdominal cavity only a mild hepatic congestion and enlargement was seen; in the thoracic cavity, a severe cardiomegaly was present while the lungs showed only disseminated red spots and lobular consolidation. The trunk of the pulmonary artery was enlarged while the aorta was reduced in size; both caudal and cranial vena cava were turgid. The heart breadth was 13 cm along the atrioventricular sulcus and 17.5 cm in length from apex to the base of cranial vena cava, being 11.5 cm the distance between atrioventricular sulcus and apex (ventricular length) and 6 cm from the atrioventricular sulcus to the base (atrial length) (Figure 2). On the cut surface the thickness of right atrial free wall and interventricular septum was 24 mm while left ventricular free wall was 20 mm thick. The right atrium was severely enlarged and in the interatrial septum a large 3 × 2.5 cm septal defect was present in the oval fossa; the defect was partially covered by a flap with fishnet appearance that protruded in an aneurismatic manner into left atrium (ostium secundum type ASD) (Figure 2). The tricuspidal ostium was dilated and the valvular leaflets thickened and distorted; the right ventricle was very enlarged with prominent trabeculae carnae; two small 4 × 3 mm paired VSD were present just beneath the septal tricuspidal leaflet. A smaller muscular VSD was present near the apex of the heart, partially obscured by redundant trabeculae carnae. The aortic inlet was evident just beneath the anterior leaflet of tricuspid valve between the leaflet and the supraventricular crest (Figure 2). Left ventricle was hypoplastic, atrioventricular ostium reduced and bicuspid leaflets distorted and thickened with a small valvular hematocyst. Ductus arteriosus was normally contracted. Histological examination showed mild multifocal suppurative embolic pneumonia and diffuse congestion of liver and kidney. No histologic anomaly was present in myocardial tissue.

## 3. Discussion

Abnormal ventriculoarterial connections are complex congenital cardiac malformations reported in humans and domestic animals [16,17,18,19,20,21,22]. These anomalies can present a wide spectrum of anatomic abnormalities: the great arteries can be connected to the inappropriate ventricles (transposition of the great vessels); aorta and pulmonary artery can arise from the left, the right, or a common ventricle (DORV, right ventricle or indeterminate ventricle, respectively); the great arteries can emerge from the base of the heart as a common trunk (single common arterial trunk) or pulmonary artery can be absent and only the aorta is identified (solitary arterial trunk); aorta can be shifted slightly to the right and arise directly above a VSD with concomitant pulmonic stenosis and RV hypertrophy (tetralogy of Fallot) [17,18,19,20,21,22].

Double-outlet right ventricle, characterized by both arterial trunks emerging from the right ventricle, has been reported in humans with an incidence of 1–3% of all CHD [23,24]. Commonly, DORV is classified in different types considering the localization of VSD, the position of the great vessels respect to VSD, the spatial relationship between the great vessels themselves, the presence of pulmonic/aortic outflow tract obstruction and the detection of other concomitant cardiac defects [18,23,25,26]. The incidence of DORV in domestic animals is rare and only few case reports have been reported in cats, dogs, horses, pigs, alpacas, and calves [6,7,21,27,28,29,30,31]. This complex congenital cardiac malformation has been only described in Angus, Brangus, Herford and Holstein breeds [6,7,8,9,10]. To the best of our knowledge, the present report describes for the first time this complex cardiac malformation in a Chianina calf.

The etiology of congenital cardiac anomalies is yet to be established in animals: heritability, maternal or fetal infection, fetal anoxia secondary to placental insufficiency and metabolic dysfunction have been reported as factors that can contribute to the development of congenital cardiac malformations [20,32]. Heritability has been reported in the Limousine breed affected by VSD [20]. In humans, chromosomal anomalies, single-gene abnormalities and teratogenic exposures can cause DORV, and specific chromosomal disorders allow to distinct this complex congenital cardiac defect in different anatomic subtypes [23]. However, how genetic disorders or teratogenic exposures can result in DORV remains unclear [23]. In the present report the cause of DORV is unknown.

In cattle, a diagnosis of CHD is suspected when clinical signs as weakness, respiratory distress and heart murmur are present [2,3,4,20,32]. Frequently, a history of failure to thrive and/or respiratory disease unresponsive to appropriate therapy is also reported [2,33,34]. Tachypnea, cyanotic mucous membranes and right-sided systolic heart murmur detected in the calf of this report were in accordance with what has been previously described in cattle affected by DORV [6,7,8,9]. Tachypnea and cyanosis, especially evident after excitement, were considered as result of anatomic right-to-left shunting of blood in the present calf, while the right-sided systolic heart murmur was due to tricuspid regurgitation secondary to annular dilatation from right heart enlargement.

Anatomically, DORV must be associated with any of the right to left communications (ASD, VSD, patent ductus arteriosus (PDA) or a combination of the former) to allow arterial and venous blood to mix and deliver oxygen to peripheral tissues. In the reported cases of bovine DORV, a various combination of shunting was present, the most common being represented by VSD that were either large or double, accounting for the shunting of blood [7,8,9,10]. DORV in the absence of VSD is very rare both in humans and animals and accounts for only one of the reported bovine cases [6]. In the present case, three muscular VSD were present, but due to the small size and the presence of large trabeculae carneae that obstructed the opening of the defects, it is reasonable to consider the ASD as the main source of shunting of the blood. Atrial septal defect was present in three of the reported bovine cases (ostium secundum-type ASD); in the remainder cases a patent foramen ovale allowed interatrial communication, being the only communication in one case; in the present case a large aneurysmal-like ostium secundum-type ASD was present as the main communication. Other cardiac anomalies are reported in association with DORV (PDA, persistent right aortic arch, aberrant origin of subclavian artery) the commonest being pulmonary stenosis; in the present case pulmonary valve and artery were normal, as was aortic artery. It is probable that the same heterogeneity of phenotypic presentations of DORV described in humans could also present in bovine and other species, affecting prognosis and postnatal survival time.

Echocardiography is a safe and noninvasive imaging technique that can be useful to confirm or rule out the presence of CHD in cattle [3,4,34]. Moreover, this diagnostic tool is readily available and can be performed in veterinary hospitals as well as in farms. In the calf of this report, echocardiography provides a definitive diagnosis of DORV associated with interatrial and interventricular septal defects. Antemortem distinction between DORV from other abnormal ventriculoarterial connections such as the complete transposition of the great vessels or tetralogy of Fallot can be difficult. Common embryologic origin of these defects can cause a misdiagnosis during echocardiographic examination. A definitive diagnosis of DORV is made when aorta and pulmonary artery emerge as a distinct conus from the right ventricle and no fibrous continuity between the aortic and mitral valves can be demonstrated [7,9]. In the calf of this report, the visualization of both great vessels leaving the right ventricle in parallel alignment with the tricuspid valve, suggested a dual outlet right ventricle; moreover, the anatomic continuity between the aortic and mitral valves cannot be visualized. Finally, different echocardiographic views and modalities also allowed to visualize a double communication (interatrial and interventricular) between the right-sided and left-sided cardiac chambers. All these echocardiographic findings were confirmed by post-mortem examination.

Congenital cardiac malformations have a guarded to poor prognosis, can be associated with stunted growth and poor productive performance, and often leading to sudden death [4,35]. The calf of this report was affected by a complex CHD without specific treatments available and a progressive worsening of the severity of the clinical signs were reported by the owner. In literature, most of animals affected by DORV presented a survival time <1 year of age, as they succumb suddenly or were euthanized because of progressive worsening of the severity of the clinical signs [6,7,9,22,28,30].

## 4. Conclusions

This case report describes a rare complex congenital cardiac malformation in a Chianina calf. To the authors’ knowledge, a similar congenital cardiac anomaly has never been reported in the Chianina breed. The clinical, ultrasonographic and pathological findings presented in this report suggest that DORV should be considered as a differential diagnosis in calves presenting with a cyanotic CHD also in the Chianina breed.

## Data Availability

The data presented in this study are available in the article.
